# Mandatory and seasonal vaccination against COVID-19: Attitudes of the vaccinated people in Serbia

**DOI:** 10.1017/S0950268823000614

**Published:** 2023-04-28

**Authors:** Verica Jovanovic, Marija Milic, Jelena Dotlic, Smiljana Cvjetkovic, Vida Jeremic Stojkovic, Natasa Maksimovic, Maja Sekulic, Tatjana Gazibara

**Affiliations:** 1 Department of Immunization, Institute of Public Health of Serbia ‘Dr Milan Jovanovic Batut’, Belgrade, Serbia; 2 Department of Epidemiology, Faculty of Medicine, University of Pristina temporarily seated in Kosovska Mitrovica, Kosovo, Serbia; 3 Clinic for Obstetrics and Gynecology, Clinical Center of Serbia, Belgrade, Serbia; 4 Faculty of Medicine, University of Belgrade, Belgrade, Serbia; 5 Department of Humanities, Faculty of Medicine, University of Belgrade, Belgrade, Serbia; 6 Institute of Epidemiology, Faculty of Medicine, University of Belgrade, Belgrade, Serbia

**Keywords:** COVID-19, mandatory vaccination, seasonal vaccination, third vaccine dose

## Abstract

The aim of our study was to examine the position of vaccinated people regarding the proposal for mandatory and seasonal vaccination against COVID-19 in Serbia. A cross-sectional study was conducted in a sample of people who came to receive a third dose of COVID-19 at the Institute of Public Health of Serbia in September and October 2021. Data were collected by means of a sociodemographic questionnaire. The study sample comprised 366 vaccinated adults. Factors associated with the belief that vaccination against COVID-19 should become mandatory were being married, being informed about COVID-19 from TV programmes and medical journals, trust in health professionals, and having friends affected by COVID-19. In addition to these predictors, factors associated with the belief that COVID-19 vaccination should become seasonal were being older, consistently wearing facemasks, and not being employed. The results of this study highlight that trust in information delivery, evidence-based data, and healthcare providers may be a major driver of mandatory and seasonal vaccine uptake. A careful assessment of the epidemiological situation, the capacity of the health system, and the risk–benefit ratio is needed in order to introduce seasonal and/or mandatory vaccination against COVID-19.

## Introduction

In Serbia, the COVID-19 pandemic emerged in March 2020. To control the propagation of the virus in human population, the Serbian government imposed a 2-month-long curfew. Older people were banned from leaving their homes, while movement of people in the younger age groups was restricted [1]. After the end of curfew, Serbia experienced a rise in new COVID-19 cases, which resulted in strict measures to limit social interactions and use of public spaces. Mass vaccination against COVID-19 began in February 2021 when four vaccines from different manufacturers were freely available to all [2]. Despite considerable efforts to increase the vaccination coverage, which, among others, included financial rewards [3], approximately one half of 7 million Serbian citizens received at least one dose of COVID-19 vaccines. Subsequently, several epidemic waves were clearly distinguishable since the beginning of the epidemic [4] wherein more than 2 million people got affected and more than 16,000 people died.

Compelling evidence suggests that vaccination against COVID-19 is remarkably effective in preventing the need for hospital treatment and premature deaths [5] and lowering the costs associated with the delivery of healthcare services [6], which is especially important in middle-income countries like Serbia. The analysis of mortality and immunisation status has found higher mortality in countries with lower vaccination coverage [7]. Thus, the benefits of vaccination as an effective means to control the spread and mitigate the consequences of COVID-19 are expectedly high.

Vaccine hesitancy, that is, delay and/or refusal to receive the vaccine despite their accessibility, has been recognised as a major threat [8]. Because of this, some countries were urged to introduce mandatory vaccination for healthcare workers and/or workers in long-term care facilities [9], while Austria was the first country in Europe to mandate COVID-19 immunisation for citizens over age 14 years [10]. In Serbia, however, vaccination has been voluntary and free of charge throughout the pandemic. Still, periodical resurgence of COVID-19 cases has initiated public debates as to whether vaccination should become seasonal (i.e. administered every year, typically in mid-autumn – which corresponds to the month of November in the northern hemisphere – when the incidence of respiratory infections in the population rises) or even mandatory, because the level of antibodies to COVID-19 gradually declines over 3–6 months after vaccination. So, periodic booster doses may be needed to maintain its effectiveness against severe COVID-19 forms [11, 12].

Bearing in mind the suboptimal vaccination coverage with one vaccine dose in Serbia and the ongoing transmission of COVID-19 in the population, the aim of this study was to examine the position of vaccinated people regarding the proposal for mandatory and seasonal vaccination against COVID-19.

## Methods

This cross-sectional study was conducted at the Institute of Public Health of Serbia (located in the capital city Belgrade), which is the chief organisation for pandemic prevention and control in Serbia. It is also one of the main points to receive vaccines against COVID-19. Adults (>18 years) who came to receive a third dose of COVID-19 vaccine in September and October 2021 were invited to participate in this study. Serbia has universal access to healthcare and vaccination against COVID-19 for all. Vaccination booths were open seven days per week from 8 a.m. to 4 p.m. and no prior appointment was required. Mass vaccination in Serbia began in February 2021. The administration of the third vaccine dose began in September 2021.

The sample size was calculated using an online tool (http://www.raosoft.com/samplesize.html) based on the adult population in Belgrade of approximately 1,000,000 people, response distribution of 70% (it was empirically expected that about 70% of the vaccinated people have a positive opinion about mandatory vaccination), margin of error of 5% and confidence interval of 95%. The calculated sample size was 322. Because of possible failure of participation, we increased the sample size by 15%.

This study was approved by the ethics committee of the Institute of Public Health of Serbia. Participation was voluntary and anonymous. Prior to enrolment, respondents were asked to provide consent for participation.

### Data collection

A questionnaire was used to collect data anonymously. It was derived from a previous qualitative study that focused on identifying a spectrum of motives of people to receive and preferences of COVID-19 vaccines [2]. In this study, a questionnaire was constructed to further explore the attitudes and preferences of people regarding COVID-19 vaccines.

First, sociodemographic characteristics of participants were examined: gender, age, education level (primary, secondary, higher), employment status (employed, unemployed, retired, student), and marital status (married/coupled, single, widowed, divorced). Next, lifestyle and behaviours were tested: current smoking status (smoker – a person who currently smokes at least one cigarette per day versus non-smoker, including former smokers), alcohol intake (never/rarely, several times per month, several times per week), recreational physical activity (fast-paced walking, swimming, aerobic, jogging, cycling, etc.), and wearing facemasks (never/seldom, only outside, in crowded spaces, always both indoors and outdoors). Participants were asked to assess their risk for contracting COVID-19 (low, medium, high). They were also asked to circle any of the listed chronic illnesses they were suffering if clinically verified (cardiovascular, pulmonary, metabolic, neurologic, rheumatologic, gastrointestinal, renal, or malignancy).

The following set of items in the questionnaire examined the source of information about COVID-19 for our participants (TV, print media, internet, YouTube, social media, Spotify, family physician, family and friends, people around participants, and scientific medical journals). Next, we asked the participant to share their experiences with COVID-19 by identifying people in their social circle who were affected (parents, partners, children, siblings, other relatives, friends) and whether they themselves were affected and needed hospital admission.

Next, participants were asked whether they trusted the following institutions and individuals: government, healthcare workers, teachers, family, friends and neighbours, army, religious leaders, and celebrities. Responses in this segment were graded on a Likert-type scale: 1 – not at all; 2 – a little; 3 – somewhat; 4 – quite a bit; 5 – a lot. Responses were transformed to binary values so that grades 1–3 were treated at negative answers (no) and values 4 and 5 as positive answers (yes).

The questionnaire is provided in Supplementary Material.

### Study outcomes

Participants were asked ‘Do you think that vaccination against COVID-19 should become mandatory?’ and ‘Do you think that vaccination against COVID-19 should become seasonal (administered every year in autumn before respiratory infections become more frequent similar to influenza immunisation)?’ The answers were binary in order to make a clear distinction in polarising attitudes and to classify people based on their general opinion. This helped us to fine-tune the analysis and identify several explanatory variables associated with this binary opinion.

### Data analysis

The data collected were described using count and percentage, median and interquartile range. Differences in categorical variables were tested using the Chi-squared test, Chi-square linear-by-linear association, or Fisher’s exact test (depending on the number of categories per variable and count per cell). Differences in continuous variables were tested using the Mann–Whitney test, because their distribution deviated from normal. To test the associations between collected variables and positive attitudes towards mandatory and seasonal vaccination, all data were classified according to logical groups presented in the questionnaire. Two sets of logistic regression models were constructed: one where the dependent variable (*outcome*) was a binary response to the question, ‘Should COVID-19 vaccination be mandatory?’ (*yes* vs. *no*) and the other where the dependent variable (*outcome*) was a binary response to the question, ‘Should COVID-19 vaccination be seasonal?’ (*yes* vs. *no*).

The independent variables were classified into the following groups: (1) ‘demographic and lifestyle model’ where all demographic and health-related characteristics were entered, including self-assessed risk for COVID-19; (2) ‘sources of information model’ where all the examined sources of information about COVID-19 were entered; (3) ‘COVID-19 experiences model’ where all reported COVID-19 cases from participants’ social circle were entered, including participants’ own experience with COVID-19; (4) ‘trust in institutions and individuals model’ where entities that participants found trustworthy were entered. All models were adjusted for gender and age.

Data analysis was carried out using the Statistical Package for Social Sciences, version 20. The limit of statistical significance was *p* = 0.05

## Results

A total of 412 participants were invited to participate. Of those, 46 people declined participation. The study sample comprised 366 participants (response rate 88.8%) with the majority being women (64.8%) aged on average 41.6 ± 15.5 years. Demographic characteristics, sources of information, COVID-19-related experiences, and trust in institutions and individuals according to the opinion that COVID-19 vaccination should become mandatory as well as seasonal are presented in Table 1. Participants who were in favour of COVID-19 vaccine becoming mandatory or seasonal were older, were married, had verified chronic illnesses, assessed their risk for contracting COVID-19 as higher, and more often wore facemasks (Table 1). These individuals more often received information about COVID-19 from TV, print media, and medical journals. Also, their parents, siblings, and they themselves were less often affected by COVID-19, but their partners, children, and friends were more often affected with COVID-19 compared to people who opposed mandatory and seasonal vaccination. People who were in favour of mandatory and seasonal vaccination trusted more the government, healthcare workers, and teachers (Table 1).

**Table 1.** Participants’ data: demographic characteristics, sources of information, COVID-19-related experiences and trust in institutions and individuals (*N* = 366)

	Mandatory vaccination			Seasonal vaccination		
Variable	Yes*N* = 237*n* (%)	No*N* = 129*n* (%)	*p*	Yes*N* = 230*n* (%)	No*N* = 136*n* (%)	*p*
Gender						
Male	84 (35.4)	54 (41.9)	0.226	84 (36.5)	54 (39.7)	0.508
Female	153 (64.6)	75 (58.1)		146 (63.5)	82 (60.3)	
Median age (interquartile range)	43.0 (25.0)	39.0 (23.0)	**0.032**	43.5 (28.7)	36.0 (21.0)	**0.001**
Marital status						
Married/coupled	139 (58.6)	56 (43.4)	**0.005**	140 (60.9)	54 (39.7)	**0.001**
Other (single, divorced, widowed)	98 (41.4)	73 (56.6)		90 (39.1)	82 (60.3)	
Education level						
Primary/secondary	183 (77.2)	97 (75.2)	0.663	45 (20.4)	39 (28.7)	0.066
Higher	54 (22.8)	32 (24.8)		185 (79.6)	97 (71.3)	
Employment status						
Employed	162 (68.4)	88 (68.2)	0.978	151 (65.7)	99 (72.8)	0.169
Other (unemployed, retired, student)	75 (31.6)	41 (31.8)		79 (34.2)	37 (24.2)	
Current smoking status						
Smoker	61 (25.7)	29 (22.5)	0.489	60 (26.1)	30 (22.2)	0.408
Alcohol drinking, yes	198 (83.5)	106 (82.2)	0.738	190 (82.6)	113 (83.7)	0.788
Practice of some form of recreational activity, yes	192 (81.0)	105 (81.4)	0.929	188 (81.7)	108 (80.0)	0.682
Having chronic illnesses, yes	126 (53.2)	60 (46.5)	0.224	132 (57.4)	54 (40.0)	**0.001**
Self-assessed risk for COVID-19						
Low	69 (29.1)	42 (32.6)	0.085	67 (28.9)	46 (33.9)	**0.041**
Medium	115 (48.5)	70 (54.2)		113 (49.1)	73 (54.1)	
High	53 (22.3)	17 (13.2)		50 (21.9)	17 (12.0)	
Wearing masks						
Never/seldom	11 (4.7)	8 (6.2)	**0.018**	9 (3.9)	10 (7.4)	**0.007**
Only indoors	80 (33.9)	57 (44.2)		78 (33.6)	59 (43.7)	
Whenever it is crowded	69 (29.2)	35 (27.1)		69 (30.1)	35 (25.9)	
Always (indoors and outdoors)	77 (32.2)	29 (22.5)		74 (32.3)	31 (23.0)	
Sources of information about COVID-19						
TV programme, yes	125 (52.7)	56 (43.4)	0.088	125 (54.3)	56 (41.5)	**0.018**
Print media, yes	54 (22.8)	26 (20.2)	0.561	58 (25.2)	22 (16.3)	**0.047**
Internet, yes	113 (47.7)	64 (49.4)	0.724	111 (48.3)	66 (48.9)	0.908
Social networks, yes	7 (3.0)	6 (4.7)	0.406	36 (15.7)	25 (18.5)	0.479
YouTube, yes	2 (0.8)	1 (0.8)	0.944	6 (2.8)	7 (5.2)	0.203
Spotify, yes	37 (15.6)	19 (14.8)	0.846	3 (1.3)	0 (0.0)	0.299
Family physician, yes	74 (31.3)	44 (34.1)	0.573	34 (14.8)	21 (15.7)	0.819
Family and friends, yes	37 (15.6)	24 (18.6)	0.463	72 (31.3)	46 (34.1)	0.585
People around me, yes	34 (14.3)	21 (16.3)	0.621	29 (12.6)	26 (19.3)	0.086
Medical journals, yes	100 (42.2)	35 (27.1)	**0.004**	101 (43.9)	33 (24.4)	**0.001**
Experiences with COVID-19						
Parents had COVID-19, yes	44 (18.6)	37 (28.7)	**0.026**	37 (16.1)	44 (32.6)	**0.001**
Partner had COVID-19, yes	40 (16.9)	24 (18.6)	0.678	41 (17.8)	22 (16.3)	**0.001**
Children had COVID-19, yes	33 (13.9)	13 (10.1)	0.289	35 (15.2)	10 (7.4)	**0.028**
Siblings had COVID-19, yes	36 (15.2)	29 (22.5)	0.081	32 (13.9)	33 (24.4)	**0.011**
Cousins had COVID-19, yes	48 (20.3)	23 (17.8)	0.575	44 (19.1)	27 (20.0)	0.839
Friends had COVID-19, yes	214 (90.3)	103 (79.8)	**0.017**	20.5 (89.1)	111 (82.8)	0.285
I had COVID-19, yes	59 (24.9)	50 (38.3)	0.377	62 (26.9)	47 (34.8)	0.191
I was hospitalised for COVID-19, yes	4 (1.7)	4 (3.1)	**0.005**	5 (2.2)	3 (2.2)	0.999
Trust in institutions and individuals*							
Trust in government	1.0 (2.0)	1.0 (1.0)	0.401	2.0 (2.0)	1.0 (1.0)	**0.033**
Trust in healthcare workers	4.0 (1.0)	4.0 (2.0)	**0.001**	5.0 (1.0)	4.0 (2.0)	**0.001**
Trust in teachers	3.0 (2.0)	3.0 (2.0)	0.106	3.0 (2.0)	3.0 (2.0)	**0.005**
Trust in family	5.0 (1.0)	5.0 (1.0)	0.239	5.0 (1.0)	5.0 (1.0)	0.053
Trust in friends and neighbours	3.0 (1.0)	4.0 (1.0)	0.908	4.0 (1.0)	3.0 (1.0)	0.417
Trust in army	3.0 (3.0)	3.0 (2.0)	0.737	3.0 (3.0)	3.0 (2.0)	0.727
Trust in religious leaders	2.0 (2.0)	2.0 (3.0)	0.357	2.0 (2.0)	3.0 (3.0)	0.138
Trust in celebrities	1.0 (2.0)	1.0 (2.0)	0.811	1.0 (2.0)	1.0 (2.0)	0.554

* Median (interquartile range), range of answers from 1 – not at all to 5 – quite a lot. Bolded values denote statistically significant difference

**Figure 1.**
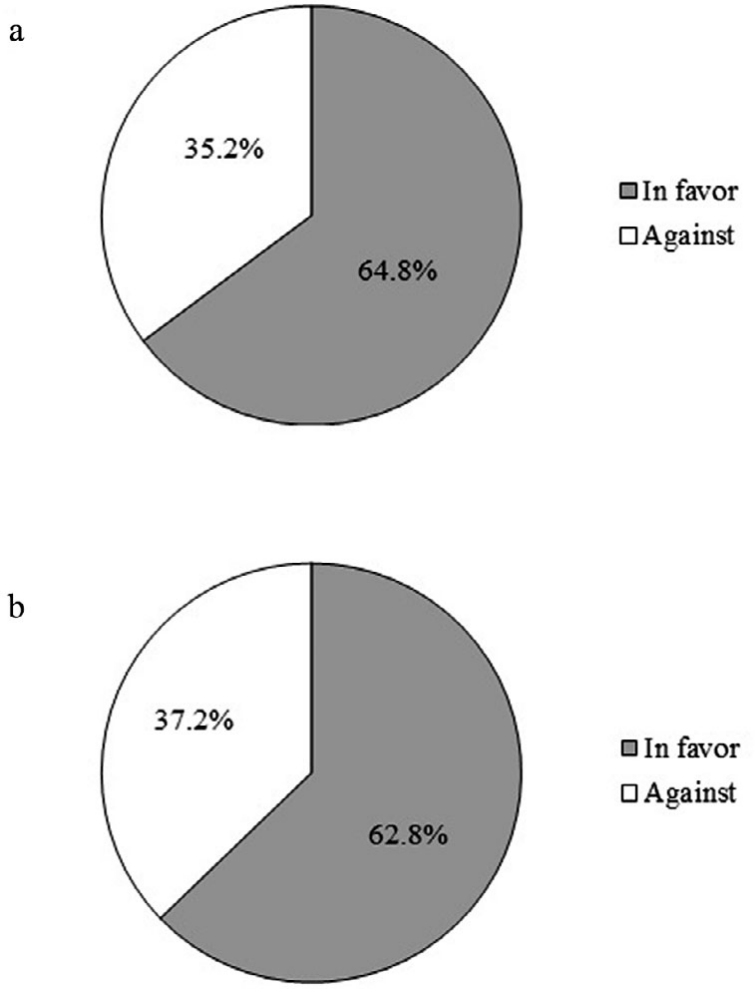
Distribution of study respondents according to the opinion that (a) COVID-19 vaccination should be mandatory and (b) COVID-19 vaccination should be seasonal.

The distribution of opinions about mandatory and seasonal vaccination in presented in Figure 1.

### Factors associated with mandatory COVID-19 vaccination

Tables 2–5 display the results of logistic regression models focusing on factors associated with the belief that COVID-19 vaccination should be mandatory. The ‘demographic and lifestyle model’ suggests that being married was associated with the opinion that COVID-19 vaccination should be mandatory (Table 2).

**Table 2.** Demographic, lifestyle and COVID-19-related risks associated with the opinion that COVID-19 vaccination should be mandatory or seasonal

Variable	Opinion							
	Mandatory				Seasonal			
	Odds ratio	95% confidence interval		*p*	Odds ratio Lower bound	95% confidence interval		*p*
		Lower bound	Upper bound			Lower bound	Upper bound	
Gender: male vs. female	1.23	0.76	1.98	0.405	1.10	0.67	1.79	0.705
Age	1.01	0.99	1.02	0.395	1.02	1.01	1.04	**0.015**
Marital status: married/coupled vs. other	1.92	1.19	3.10	**0.007**	1.54	0.95	2.51	0.079
Education level: higher vs. other	1.29	0.74	2.01	0.413	1.73	0.97	2.62	0.217
Employment status: employed vs. other	0.84	0.51	1.38	0.499	0.57	0.34	0.95	**0.032**
Smoking: Smoker vs. non-smoker	0.99	0.82	1.19	0.907	0.99	0.82	1.19	0.937
Alcohol drinking	1.13	0.89	1.44	0.307	1.24	0.97	1.60	0.089
Physical activity	1.13	0.62	2.06	0.679	1.24	0.68	2.27	0.481
Having chronic illnesses: yes vs. no	1.10	0.67	1.81	0.714	1.47	0.90	2.42	0.127
Self-assessed risk for COVID-19	1.15	0.81	1.63	0.426	1.16	0.81	1.65	0.418
Wearing masks	1.28	0.99	1.65	0.062	1.35	1.04	1.75	**0.024**

Bold values suggest statistical significance.

The ‘sources of information model’ shows that receiving information about COVID-19 from TV programmes and medical journals was associated with having a positive opinion about the COVID-19 vaccine becoming mandatory (Table 3).

**Table 3.** Sources of information associated with the opinion that COVID-19 vaccination should be mandatory or seasonal

Variable	Opinion							
	Mandatory vaccination				Seasonal vaccination			
	Odds ratio	95% confidence interval		*p*	Odds ratio Lower bound	95% confidence interval		*p*
		Lower bound	Upper bound			Lower bound	Upper bound	
Gender: male vs. female	1.28	0.80	2.05	0.300	1.19	0.73	1.93	0.479
Age	1.01	0.99	1.03	0.155	1.03	1.01	1.04	**0.002**
TV programme: yes vs. no	1.75	1.05	2.91	**0.031**	1.85	1.10	3.10	**0.019**
Print media: yes vs. no	1.02	0.56	1.86	0.944	1.55	0.82	2.91	0.176
Internet: yes vs. no	0.84	0.51	1.38	0.500	0.84	0.52	1.40	0.512
Social networks: yes vs. no	0.90	0.47	1.70	0.739	0.96	0.50	1.85	0.903
YouTube: yes vs. no	0.52	0.16	1.72	0.286	0.38	0.11	1.36	0.137
Spotify: yes vs. no	1.15	0.09	14.75	0.912	N/A			
Family physician: yes vs. no	1.03	0.54	1.96	0.937	0.88	0.46	1.71	0.715
Family and friends: yes vs. no	0.78	0.45	1.33	0.356	0.84	0.48	1.46	0.540
People around me: yes vs. no	1.03	0.52	2.05	0.928	0.67	0.33	1.34	0.257
Medical journals: yes vs. no	2.16	1.32	3.52	**0.002**	2.79	1.68	4.63	**0.001**

Bold values suggest statistical significance; N/A, not applicable.

Further, the ‘COVID-19 experiences model’ found that people whose friends had COVID-19 and people who were not previously affected by COVID-19 were more likely to believe that COVID-19 vaccination should be mandatory (Table 4).

**Table 4.** COVID-19-related experiences associated with the opinion that COVID-19 vaccination should be mandatory or seasonal

Variable	Opinion							
	Mandatory				Seasonal			
	Odds ratio	95% confidence interval		*p*	Odds ratio Lower bound	95% confidence interval		*p*
		Lower bound	Upper bound			Lower bound	Upper bound	
Gender: male vs. female	1.18	0.74	1.87	0.482	1.06	0.67	1.69	0.791
Age	1.01	0.99	1.03	0.342	1.02	1.01	1.04	**0.014**
Parents had COVID-19: yes vs. no	0.67	0.37	1.20	0.181	0.57	0.32	1.01	0.053
Partner had COVID-19: yes vs. no	0.98	0.51	1.89	0.947	1.01	0.52	1.96	0.974
Children had COVID-19: yes vs. no	1.45	0.65	3.25	0.364	1.39	0.60	3.20	0.441
Siblings had COVID-19: yes vs. no	0.85	0.45	1.60	0.618	0.72	0.38	1.35	0.307
Cousins had COVID-19: yes vs. no	1.15	0.65	2.05	0.627	1.06	0.60	1.87	0.846
Friends had COVID-19: yes vs. no	1.57	1.15	2.15	**0.005**	1.45	1.06	1.99	**0.020**
I had COVID-19: yes vs. no	0.55	0.33	0.97	**0.039**	0.78	0.44	1.37	0.390
I was hospitalised for COVID-19: yes vs. no	0.61	0.13	2.79	0.524	0.73	0.15	3.52	0.692

Bold values suggest statistical significance.

Finally, the ‘trust in institutions and individuals model’ found that people who were older and who considered healthcare workers as the most trustworthy people with regard to COVID-19 management were also more likely to think positively about mandatory vaccination (Table 5).

**Table 5.** Trust in groups and individuals associated with the opinion that COVID-19 vaccination should be mandatory or seasonal

Variable	Opinion							
	Mandatory				Seasonal			
	Odds ratio	95% confidence interval		*p*	Odds ratio Lower bound	95% confidence interval		*p*
		Lower bound	Upper bound			Lower bound	Upper bound	
Gender: male vs. female	1.40	0.873	2.25	0.163	1.16	0.70	1.91	0.560
Age	1.02	1.00	1.03	**0.029**	1.04	1.03	1.06	**0.001**
Trust in government	1.07	0.82	1.41	0.590	1.25	0.94	1.66	0.132
Trust in healthcare workers	1.62	1.24	2.12	**0.001**	1.86	1.39	2.48	**0.001**
Trust in teachers	1.07	0.84	1.36	0.587	1.23	0.96	1.59	0.103
Trust in family	1.09	0.82	1.45	0.540	1.09	0.81	1.47	0.557
Trust in friends and neighbours	0.90	0.69	1.16	0.402	1.01	0.77	1.32	0.938
Trust in army	1.04	0.80	1.34	0.785	1.02	0.78	1.33	0.887
Trust in religious leaders	0.82	0.65	1.03	0.092	0.73	0.58	0.93	**0.013**
Trust in celebrities	1.07	0.83	1.37	0.608	0.76	0.59	0.99	**0.044**

Bold values suggest statistical significance.

### Factors associated with seasonal COVID-19 vaccination

Tables 2–5 show the results from logistic regression models about factors associated with the opinion that the COVID-19 vaccine should be seasonal. The ‘demographic and lifestyle model’ suggests that being older, not being employed, and consistently wearing facemasks were associated with a positive opinion that COVID-19 vaccination should be administered seasonally (Table 2).

The ‘sources of information model’ shows that being older, and receiving information about COVID-19 from TV programmes and medical journals was associated with the belief that COVID-19 vaccine should be seasonal (Table 3).

The ‘COVID-19 experiences model’ suggests that being older and having friends affected by COVID-19 were associated with a positive opinion about seasonal vaccination (Table 4).

The ‘trust in institutions and individuals model’ found that people who were older and who considered healthcare workers as the most trustworthy people with regard to COVID-19 management were also more likely to think positively about seasonal vaccination. These people were less likely to trust religious leaders and celebrities (Table 5).

## Discussion

This study shows that about two-thirds of vaccinated people were in favour of COVID-19 vaccines becoming mandatory and seasonal. People who were married and older, who were informed about COVID-19 through TV programmes and medical journals, who had friends affected by COVID-19, but did not personally suffer from COVID-19, and who trusted healthcare workers were more likely to believe that COVID-19 vaccination should be mandatory. In addition, not participating in the workforce and regular use of facemasks were associated with the position that COVID-19 vaccination should become seasonal.

A large body of evidence explored public opinions about mandatory COVID-19 vaccination [13–19], albeit in a general population including non-vaccinated ones as well. Contrary, there are limited studies conducted on vaccinated population in the literature. This study focused solely on those who received three vaccine doses. Although the beliefs of the general public are important, we considered that the opinions of vaccinated people are even more relevant as these individuals have been responsive to measures taken by the policymakers, but still have a mind of their own. They have also been shown (by medical professionals and media) to present different and often contradictory information regarding vaccination, but this group seemed to have made decisions according to scientific data overcoming irrational fear, fake news, and conspiracy theories.

Our study shows that the majority of vaccinated people, but not all, support the notion of mandating vaccination against COVID-19. A large study in Germany reported that being vaccinated with at least one dose is predictive of having a positive opinion about the proposal of mandatory vaccination [20]. Such findings present an important piece of information for researchers, public health professionals, policymakers, and other stakeholders. Strategies to increase vaccination coverage by focusing on people who reject vaccination per se were not proven to be very successful in the Serbian setting. Therefore, policymakers should account for the attitudes of people who chose to receive the vaccine regardless of all negative information about COVID-19 vaccination. Possibly, if their opinion was taken into consideration, different population groups would be easier to reach.

Moreover, our study results point out several specific explanatory variables that are likely predictive of having such an attitude. These predictors call for a deeper understanding of people’s attitudes, especially information delivery and personal experiences. Previous investigations showed that mandatory vaccination against COVID-19 is more often considered acceptable for those working in certain professions, such as in settings with a high turnover of people. On the other hand, it may be a condition to travel and study abroad rather than a mandate for the general population or children [9, 10, 19], while some believe that COVID-19 vaccination should be mandatory for healthcare workers [18].

The high percentage of positive attitudes towards mandatory or seasonal vaccination in our study can be explained by the fact that we studied people who received all vaccine doses recommended to them by the time of survey and who therefore expressed a generally positive attitude towards vaccination. However, even in this population, one-third of participants were not in favour of mandatory and/or seasonal vaccination. In fact, many people in the general population in Serbia have conspiracy beliefs relative to COVID-19 that are coupled with poor knowledge about vaccines [21]. Even before the onset of the COVID-19 pandemic, vaccine hesitancy in Serbia was on the rise. This was corroborated by the analysis of vaccination coverage in the national immunisation programme [22] as well as with the resurgence of measles in 2017–19 [23]. As a result, in the recent years and during the COVID-19 pandemic, vaccination was heavily scrutinised by the public.

It is not surprising that older people in this study were more likely to support mandatory vaccination. Older people are considered a high-risk group for poor COVID-19 outcomes, and this has been consistently discussed in the media. They also seem to be consistently wearing facemasks and adhering to recommendations for COVID-19 prevention [24]. Having friends who were affected and not being personally affected with COVID-19 were also predictive of a positive attitude towards mandatory and seasonal vaccination. A previous study has shown that higher levels of concern were associated with having friends and family members being affected by COVID-19 [25], which corroborated our results as well.

Trust in information is one of the key challenges during the COVID-19 pandemic because of information overload [26]. People in this study favouring mandatory and seasonal COVID-19 vaccination were more likely to follow official sources such as mainstream media, evidence-based data, and healthcare professionals. Trust in healthcare providers and official sources of information is pivotal for people to adhere to recommendations about prevention [27]. To enhance public trust, another important issue is the delivery of information in clear and simple terms [28].

Beliefs about the effectiveness of COVID-19 vaccines, trust in public institutions and infringements of personal freedom are the key underlying mechanisms in efforts to change attitudes about vaccination during the pandemic [29]. Mandatory restrictions to travel, access to institutions or public spaces have been observed as the cause of stronger objection to vaccination, as well as labelled coercive and discriminatory [30, 31]. Some authors believe that mandatory vaccination could further divide the civil society and violate human rights [32]. Therefore, the introduction of mandatory vaccination should be carefully considered with respect to the organisation of the healthcare system and also the cultural format.

A limitation of this study is the notion that the observed attitudes cannot be generalised to the entire population of Serbia, but are applicable only to those who received COVID-19 vaccines. This limitation arises from the fact that vaccination coverage with at least one dose was approximately 50% in Serbia. The average age of the Serbian population is 43 years [33], which was well represented in our study sample. However, the composition of the study sample in terms of education level was skewed towards individuals with a higher level of education. According to the Statistical Office of Serbia, the majority of people in Serbia completed a secondary education (48.9%), while people who have post-secondary education account for 16.2% [34]. This composition of people with higher education attainment in a sample of vaccinated people is not surprising because of its consistent association with COVID-19 vaccine acceptance [35]. The questionnaire included items based on the data from a previous qualitative study [2]; however, it cannot be ruled out that some pieces of information relevant to the study outcomes may have been omitted. For this reason, the study is open to unobserved confounding. Because the data were collected at the same instance when participants received the vaccine, our study can only provide assumptions about causal associations and not actual causal inferences.

## Conclusion

In summary, people who received the third COVID-19 vaccine dose had mostly positive attitudes towards mandatory and seasonal vaccination against COVID-19 in Serbia. The results of this study highlight that trust in information delivery, evidence-based data, and healthcare providers may be the main driver of mandatory and seasonal vaccine acceptance. Also, some personal experiences, such as having friends affected by COVID-19 and stronger adherence to official recommendations, could play a role in the decision to mandate vaccination. A careful consideration of the epidemiological situation, capacity of the healthcare system and risk–benefit ratio is needed in order to introduce seasonal or/and mandatory vaccination against COVID-19.

## Data Availability

Dataset underlying this study is available on a reasonable request from the corresponding author.

## Supplementary material

For supplementary material accompanying this paper visit https://doi.org/10.1017/S0950268823000614. click here to view supplementary material
