# Supplementary material for: Mandatory and seasonal vaccination against COVID-19: Attitudes of the vaccinated people in Serbia
Source: Epidemiol Infect. 2023 Apr 28;151:e83. doi: 10.1017/S0950268823000614 (PMC10244631; doi:10.1017/S0950268823000614)
Supplement: Supplementary file 1 [file S0950268823000614sup001.doc]

**EPIDEMIOLOGICAL QUESTIONNAIRE ID __________**

**This questionnaire is anoymous. Please answer all the questions**

**1.** **Gender**: a) male b) female

**2. How old are you now?**  _______________

**3. Marital status:** a) single b) married/in a relationship e) divorced f) widowed

**4. Your highest education attainment is:** a) no education/did not complete primary school

b) primary school c) secondary school d) college/university

**5. Employment status:** a) unemployed b) employed c) studying d) retired

**6. Your smoking status is:** a) smoker (at least 1 cigarette per day) b) former smoker c) non-smoker

**7. Howe often do you drink alcohol beverages (**beer, wine, hard liquor**):**  a) never

b) once a month or less c) 2-4 times per month d) 2-3 times per week e) 4 or more times per week

**8. Do you practice some form of physical activity/recreational activity** (walking, aerobic, yoga, gym, gardening, cycling)**:** a) no b) yes

**9.** **Do you have any of the following illnesses verified by your doctor:**

1) diabetes (diabetes type 1 or 2) a) yes b) no

2) other metabolic diseases (thyroid etc) a) yes b) no

3) higher blood pressure (hypertension) a) yes b) no

4) cardiovascular diseases (arrhymias, infarction, angina, problems with veins) a) yes b) no

5) lung diseases (asthma, bronchitis, obstructive lung disease etc) a) yes b) no

6) rheumatological and autoimmune diseases (arthritis, lupus) a) yes b) no

7) neurologic diseases (epilepsy, myastenia gravis, etc) a) yes b) no

8) mental health problems a) yes b) no

9) other diseases (renal, gastrointestinal, liver problems etc) a) yes b) no

10) malignant tumors (cancer) a) yes b) no

**10. How do you assess your risk for catching COVID-19?** a) low b ) medium c) high

**11. How often do you wear face masks as protection from COVID-19:** a) never b) sometimes c) only inside d) whenever it is crowded e) all the time outside of my home

**12. Has anyone who you know personally had COVID-19 (circle all that applies to you):**

a) mother/father b) partner c) kids d) siblings e) grandparents f) partner’s family g) other relatives

**13.** **Has any of your friends had COVID-19:** a) no b) yes, one c) yes, more than one

**14. Have you ever had COVID 19?** a) yes b) no

**15. Have you been hospitalized for COVID 19?** a) yes b) no

**16. Do you think that vaccination against COVID-19 should become mandatory** a) yes b) no

**17. Do you think that vaccination against COVID-19 should become seasonal (administered every year)?** a) yes b) no

**18. What sources of information do you use to learn about COVID-19 and vaccines against COVID-19?** a) TV/radio b) daily printed newspapers c) Internet webpages

d) YouTube e) Spotify podcasts f) my family physician g) family and friends

h) social media i) people around me (acquaintances, neighbors) j) medical journals

**19. To what degree do you trust in the institutions and people listed below when it comes to COVID-19?**

1-not at all; 2-a little; 3-somewhat; 4-quite a bit 5-a lot.

| Government/politicians | 1 | 2 | 3 | 4 | 5 |
| --- | --- | --- | --- | --- | --- |
| Healthcare workers | 1 | 2 | 3 | 4 | 5 |
| Teachers | 1 | 2 | 3 | 4 | 5 |
| Family | 1 | 2 | 3 | 4 | 5 |
| Friends/neighbors/colleagues | 1 | 2 | 3 | 4 | 5 |
| Army | 1 | 2 | 3 | 4 | 5 |
| Religious leaders | 1 | 2 | 3 | 4 | 5 |
| Celebrities (musicians, actors, sport players) | 1 | 2 | 3 | 4 | 5 |
